# Liver Illness and Psoriatic Patients

**DOI:** 10.1155/2018/3140983

**Published:** 2018-02-06

**Authors:** Marco Fiore, Sebastiano Leone, Alberto Enrico Maraolo, Emilio Berti, Giovanni Damiani

**Affiliations:** ^1^Department of Anaesthesiological, Surgical and Emergency Sciences, University of Campania “Luigi Vanvitelli”, Naples, Italy; ^2^Department of Medicine, Division of Infectious Diseases, “San Giuseppe Moscati” Hospital, Avellino, Italy; ^3^Department of Clinical Medicine and Surgery, Section of Infectious Diseases, University of Naples Federico II, Naples, Italy; ^4^Department of Pathophysiology and Transplantation, Dermatology Unit, IRCCS Ca' Granda, University of Milan, Milan, Italy; ^5^Study Center of Young Dermatologists Italian Network (YDIN), Bergamo, Italy

## Abstract

Psoriasis is a chronic inflammatory disease of the skin affecting approximately 2% of the world's population. Systemic treatments, including methotrexate and cyclosporin, are associated with potential hepatotoxicity, due to either direct liver damage or immunosuppression or both immunomediated and a direct liver injury; therefore, treatment of patients with psoriasis poses a therapeutic challenge. The aim of this minireview is to help clinicians in the management of psoriatic patients who develop signs of liver dysfunction. To find relevant articles, a comprehensive search was performed on PubMed, EMBASE, and Cochrane with appropriate combinations of the following keywords being considered: viral hepatitis, nonalcoholic fatty liver disease, psoriasis, hepatotoxicity, drug toxicity, cholestasis, and autoimmune liver diseases.

## 1. Introduction

Psoriasis world prevalence was attested to be 125 million people, constituting a great problem in public health and so a main challenge. Psoriasis presents a geographical variation in prevalence, spacing from 0.91% in the USA to 8.5% in Norway [1] that may be due to differences in climate, genetic background, and exposome [2]. Notwithstanding higher latitudes, countries show the higher prevalence and Africa and Asia display the lower one; the estimation could be affected also by the lack of solid and cooperative archives. Psoriasis is a chronic inflammatory disease classically thought to affect only skin, but recently discovered to afflict systemically the whole body, including the gastrointestinal district. Likewise, other more celebrated systemic inflammatory diseases, psoriasis creates and maintains an inflammatory microenvironment in which proinflammatory mediators spread from lesional skin to other district insulting different and distant tissues and giving the rationale of the so-called comorbidities [3]. Coherent with previous studies, psoriasis is actually thought to have a potential and intrinsic hepatolesivity. This idea was recently supported by the first mouse-model of hepatitis in imiquimod-induced psoriasis [2]. Furthermore, an increased body of evidences suggests a possible association with the classical autoimmune hepatic disorders, such as neutrophilic cholangitis or primary cirrhosis [4]. Systemic treatments, including methotrexate (MTX) and cyclosporin (CyA), are associated with potential hepatotoxicity, due to either direct liver damage or immunosuppression or both immunomediated and a direct liver injury [5]; therefore, treatment of patients with psoriasis poses a therapeutic challenge. This minireview will briefly touch upon some points of liver involvement in psoriatic patients.

## 2. Evidence Acquisition

To find relevant articles, a comprehensive search was performed on PubMed, EMBASE, and Cochrane with appropriate combinations of the following keywords being considered: viral hepatitis, nonalcoholic fatty liver disease, psoriasis, hepatotoxicity, drug toxicity, cholestasis, and autoimmune liver diseases. Recent articles were in priority. Primary sources were meta-analyses, systematic reviews, and original articles in order to achieve the highest possible level of evidence.

## 3. Psoriasis and Liver Biochemistry Disturbance because of Psoriasis

Idiopathic liver biochemistry disturbance in psoriasis is not a nosographic entity well described in the literature. Tula et al. retrospectively reviewed 518 psoriasis patients, of these the liver biochemistry disturbance and the potential relation with the most common risk factors (obesity, diabetes mellitus, alcohol consumption, hepatotoxic medications, dyslipidemia, infectious hepatitis) were evaluated [6]. Elevation of liver enzymes was defined idiopathic in patients without an identified risk factor: 4% of mild-moderate and 8% of severe elevation of liver function tests [6]. However, in our opinion, this percentage (4–8%) cannot be interpreted as idiopathic because the authors (for the retrospective nature of the study) did not evaluate all the possible causes of hypertransaminasemia (e.g., autoimmune disorders, celiac disease, Hemochromatosis, and Wilson's disease) [6].

## 4. Disturbance Because of the Treatment of Psoriasis (Drug-Induced Liver Dysfunction)

Drug-induced liver injury (DILI) is a leading cause of emergency liver transplantation; it ranges from asymptomatic elevation of liver enzymes to acute liver failure. The elevation of liver enzymes in psoriasis patients is mainly associated with consumption of liver toxic substances (57%) followed by nonalcoholic fatty liver disease (NAFLD) (22%) [6]. Different drugs taken by patients with psoriasis are reported to be hepatotoxic. The most common antipsoriatic drugs associated with elevation of liver enzymes are MTX and Acitretin (ACIT). In most cases, liver enzyme levels are only mildly elevated [6]. MTX has been considered to be one of the main causes of elevation of liver enzymes, in psoriatic patients, for several years. It is a systemic medication and immune system suppressant, used to treat moderate-to-severe psoriasis and psoriatic arthritis (PsA). MTX is the most frequently used disease-modifying and rheumatic drugs (DMARDs) with over 70% of PsA patients still taking the drug. MTX rarely causes clinically significant hepatotoxicity and it is more common in PsA patients, instead pulmonary toxicity with MTX is found more often in rheumatoid arthritis (RA) [7]. In a retrospective study, conducted from 2000 to 2009 in a tertiary dermatology center in Malaysia, sixty-six of 710 (9.3%) patients with psoriasis were prescribed MTX throughout the 10-year period. Among them 57.6% developed deranged transaminases, with six requiring MTX withdrawal due to hepatotoxicity [8]. In a retrospective cohort review among patients of a large health maintenance organization in Israel who were diagnosed with either RA (*n* = 119) or psoriasis (*n* = 690) and who had purchased at least one dose of MTX, liver function analyses were performed serially in these patients during the follow-up. Both groups had hepatic enzyme elevation; the predisposing factors predictive of liver damage were female gender and a higher cumulative dose of MTX (hazard ratios, 1.46 and 1.07, resp., *p* < 0.001). Age, concurrent diseases, and type of disease had no influence on susceptibility to liver damage. No significant differences between psoriasis PsA and RA patients was found [9]. A previous prospective study, involving 550 RA patients and 69 PsA patients on MTX, showed that PsA patients have a higher incidence of hepatotoxicity compared to RA patients. In this study, alcohol consumption did not correlate with hepatic injury (mean 5.15 versus 6.6 alcohol units/week consumed by RA and PsA patients, resp.) [10]; the use of folate supplements in patients treated with MTX reduces the incidence of hepatotoxicity and gastrointestinal intolerance without impairing the efficacy of MTX [11]. An ethanolic extract of leaves of* Piper betle* (Paan) Linn is a promising antioxidant-mediated hepatoprotective agent in decreasing the MTX-induced toxicity. Future studies are needed to confirm the therapeutic efficacy [12]. Actually, MTX liver toxicity appears to be associated with underlying metabolic syndrome and NAFLD [13]; a recent meta-analysis showed a significant difference in moderate elevation of liver enzymes in obese patients with PsA treated with MTX versus nonobese patients [14]. Furthermore, obesity is reported to display a role in increasing the risk of liver toxicity from MTX and CsA [15]. The elevation of liver enzymes was evaluated in RA and PsA patients enrolled in the Consortium of Rheumatology Researchers of North America (CORRONA). Liver enzymes abnormalities were identified when the upper limits of normal (ULN) were either 1- or 2-fold times above: elevations > 2x ULN occurred in 1-2% of patients on MTX or leflunomide (LEF) monotherapy compared with 5% with the combination. Liver enzymes elevations were developed in 14–35% of RA/PsA patients, initiating DMARD therapy. The risks were incrementally greater in those with PsA and in those receiving MTX plus LEF [16]. Cumulative dose of MTX does not seem to be associated with a progression to liver cirrhosis [17]. Transient elastography and FibroTest could be effective noninvasive tools for monitoring the progression to liver cirrhosis in patients [18]. Recently, researchers propose to reduce the use of liver biopsy in patients with elevation of liver enzymes if transient elastography or FibroTest and Procollagen III peptide is performed. But this strategy is not validated in prospective studies [19]. MTX may be used in association with ACIT, CsA, prednisone, and antitumor necrosis factor alpha (TNF-*α*). The association of MTX with ACIT in the past it has been the object of a warning regarding the potential hepatotoxicity of the drug interactions. The ACIT and MTX combination therapy for psoriasis is well tolerated [20] and recently has shown higher effectiveness and less liver fibrosis [21]. ACIT is a synthetic retinoid used for severe extensive psoriasis; it is associated with abnormal liver function test findings and toxic hepatitis in 1.5% of patients [22]; ACIT-associated liver toxicity and apoptosis is possibly related to mitochondrial dysfunctions [23]. CsA hepatotoxicity is rare event [24]; the underlying mechanism is probably due to an oxidative stress and redox imbalance demonstrated in rat's hepatocytes [25]. Liver injury, although uncommon, has been observed in some patients treated with medications that inhibit the actions of TNF-*α* [5, 26]. Ustekinumab, an IL-12/23 blocker, is cause of an uncommon and mild liver injury. From a hepatic point of view, the drug appears safe, even in patients with preexisting liver disease and those who have developed altered liver function previously with other drugs [27]. Fructus Psoraleae (FP) is used by herbalists for the treatment of postmenopausal osteoporosis, vitiligo, and psoriasis. It is used alone, or in combination with other herbs, in some countries in the form of proprietary medicine. It is recognized as one of the emerging liver toxic substances [28]. A case of hepatitis and jaundice is associated with ingestion of Lotus-f3 submitted to a Norwegian regional pharmacovigilance center. A 56-year-old woman with PsA developed increased liver enzymes and jaundice 3 weeks after having started to take the product [29].  Table 1 shows comparison of liver toxic substances in psoriatic patients [7, 13, 16, 22, 23, 25–28, 30–32].

## 5. Psoriasis and Hepatitis B Virus Infection

Chronic hepatitis B affects about 3-4% of the world population, which results in being HBsAg positive. Notwithstanding, the number of people (around two billion) is far larger who has been exposed during his lifetime to hepatitis B virus (HBV), becoming occult carrier [33–35]. HBV infection has been directly linked with many skin disorders; however, the connection with psoriasis is indirect and it relies on the risk of HBV reactivation (HBVr) during immunosuppressive drug therapy (ISDT) [36, 37]. HBVr is not a univocal syndrome, ranging from clinically inapparent laboratory alterations to life-threatening liver injury [38]. It can occur both in patients with overt chronic HBV infection (HBsAg positive) and in occult HBV carriers [39, 40]. Literature data mostly focus on patients with solid tumors and hematological malignancies, yielding guidelines and recommendations to prevent and manage HBVr in these settings [41]. Nonetheless, HBVr may also involve patients undergoing ISDT because of inflammatory bowel disease and rheumatologic and/or dermatologic conditions [42]. Thus, screening for HBV all the patients who are about to commence an ISDT, and establishing the possible risk of HBVr, is mandatory in order to implement appropriate preventive measures [43, 44]. Antiviral prophylaxis is warranted in case of high or moderate risk of HBVr and a tight monitoring is necessary for remaining patients [43]. Unanswered questions include the exact duration of prophylaxis which usually started 2–4 weeks before the initiation of ISDT and prolonged at least for 6–12 months after the last dose of the ISDT [45]. As for subjects suffering from psoriasis, the problem arises especially when criteria for moderate-to-severe disease are met, requiring either conventional DMARDs (cDMARDs) or biological ones (bDMARDs) [46]. Among the cDMARDs, ACIT is not an immunosuppressive agent, although its potential liver toxicity limits its use in subjects with overt viral hepatitis [47, 48]. CsA is associated with a moderate risk of HBVr both in HBsAg-positive and in HBsAg-negative patients [43, 48], whereas MTX is linked with low risk of HBVr [43, 49]. Among the bDMARDs, the class encompassing the major number of active agents is represented by TNF inhibitors: the more potent ones (infliximab, adalimumab, golimumab, and certolizumab) pose a high risk of HBVr in HBsAg-positive patients and a moderate risk in occult HBV carriers; the less potent ones (etanercept) imply a moderate risk in the first group and even lower in the latter [43]. TNF inhibits viral replication and elicits T-lymphocyte cytotoxic response: thus the rationale explained the risk of HBVr carried by anti-TNF drugs [50, 51]. HBVr as a consequence of anti-TNF agents has been mainly reported in patients suffering from diseases different from psoriasis, such as inflammatory bowel disease and rheumatic disorders [52]. Unfortunately, data regarding use of TNF inhibitors and risk of HBVr specifically in psoriatic patients are derived from small series, often retrospective, not seldom including both subjects with only dermatological manifestations and individuals with PsA [53–55]. About HBsAg-positive patients, data come from very limited experiences with regard to sample size [49, 53]. In the most recent study, recruiting 10 patients treated with adalimumab (all with detectable viremia at baseline apart from one), no HBVr was observed; all of them were under antiviral prophylaxis [56]. In smaller series, mainly focusing on patients previously classified as “inactive carriers” and currently defined as with a “HBeAg negative chronic HBV infection” (positivity to anti-HBe, HBV DNA usually <2,000 IU/ml but sometimes fluctuating slightly over this threshold, however not >20,000 IU/ml), HBVr was observed only when no antiviral prophylaxis had been adopted [33]. In detail, Cho et al. described 3 cases of reactivation out 7 patients under etanercept [57]. There is just one case describing the use of an anti-TNF agent (infliximab) in a patient with concomitant diffuse psoriasis and chronic active HBV infection: the sequential use of lamivudine and entecavir was successful in preventing HBVr and even achieving viremia negativization [58]. Other bDMARDs have targets such as IL-17A (secukinumab) and IL-12/IL-23 (ustekinumab) [59]. In general, these cytokine-based therapies are deemed to pose a moderate risk of HBVr regardless of HBsAg status [46]. IL-17A has role in the inflammatory process accompanying chronic B hepatitis [60]. Clinical trials on secukinumab showed no increased risk of HBVr [61]. In conclusion, although evidence does not rely on high-quality studies, all patients with psoriasis who are about to undergo an ISDT need to be screened for HBV; the decision to start a prophylaxis depends on the risk of viral reactivation, which is higher in HBsAg-positive patients treated with anti-TNF agents as well as ustekinumab [62].  Table 2 summarizes the risk gradient of HBVr with different drug [45, 48, 63, 64].

## 6. Psoriasis and Hepatitis C Virus Infection

Hepatitis C virus (HCV) infection affects about 200 million people worldwide, being a major health problem [65]. HCV infection is associated with a high spectrum of extrahepatic disorders, including dermatological manifestations such as Sicca syndrome and lichen planus [66]. Some reports suggest a link between HCV and psoriasis as well [67]. In a case-control study, matching with a 1 : 2 ratio and involving 12,502 subjects, the prevalence of HCV psoriatic patients was twofold compared with the control group (1.03% versus 0.56%, *p* = 0.001); at multivariate analysis, psoriasis was associated with HCV and not with HBV, although also HBV was more frequent in the group of cases [68]. Although the pathogenesis of psoriasis remains not fully elucidated, an increased body of evidences showed a possible infectious trigger in genetically susceptible patients [69, 70]. Traditionally guttate form of psoriasis is believed to be triggered by infections, especially* Streptococcus* related [71]; however also recent studies on HCV-positive psoriatic patients start to extend the concept of infectious trigger also to other psoriasis subtypes such as plaque one [1]. Imafuku et al. reported that HCV infection contributes to develop late onset psoriasis and that HCV-positive patients have a double risk of psoriasis than uninfected ones [72]. Albeit the role of HCV as trigger in psoriasis is well known, few data are present about the role of HCV after psoriasis development. Chun et al. found increased mRNA levels of cathelicidin, Toll-like receptor (TLR)-9 and IFN-*γ* in both lesional and nonlesional skin of HCV-positive patients with psoriasis compared to HCV-negative psoriatic patients. These data, together with an increased level of IFN-*γ* in lesional skin than in nonlesional one in HCV-positive psoriatic patients, may address to a key role of HCV also in maintaining and amplify psoriasis inflammatory pathway. The proposed theory that HCV implement the expression of cathelicidin and IFN-*γ* in keratinocytes upon injury stimuli and activate Plasmacytoid dendritic cells to produce IFNs and finally initiate and maintain a Th1/Th17 inflammatory response in the skin, capable of developing psoriasis [73]. Likewise, also psoriasis can contribute to chronicize HCV infection. Despite its role in autoimmune diseases, such as psoriasis, IL-17 is also implicated in privileging the evolution from acute to chronic phase of HCV infection. In particular, the acute phase is lack of expansion of either CD4+ or CD8+ T cells producing IL-17, in contrast with chronic HCV patients that display statistically more Th17 compared to peripheral blood [74]. Beyond the controversial issue of a causal relationship, especially in areas with moderate-high HCV endemicity the concrete problem is how to manage patients suffering from psoriasis and with concomitant HCV infection [75]. One point to be addressed is the risk of acute exacerbation and/or reactivation of chronic HCV infections in patients undergoing an ISDT. Actually, the magnitude of the problem is not as relevant as for HBV [76]. Moreover, the definition of these entities is not universally standardized: however, in the setting of cancer patients, acute exacerbation was defined as a 3-fold or greater increase in serum ALT level, whereas reactivation was defined as an increase in viremia of at least 1 log^10^ IU/ml [77]. The aforementioned systematic review found HCV reactivation only in 3 out 97 patients with psoriasis under bDMARDs treatment, without evidence of hepatitis exacerbation (3.1%) [78]. Among bDMARDs, most data concern TNF antagonists: in HCV patients with psoriasis [79], their use appears safe, although sporadic cases of hepatocellular carcinoma (HCC) have been reported [49]. This occurrence affected cirrhotic patients: considering that cirrhosis by any cause is itself a remarkable prooncogenic risk factor, it is difficult to establish the role played by TNF inhibitors, which, however, should be used with caution in psoriatic patient with advanced liver disease [49, 79]; cDMARDs seem safe in HCV patients with psoriasis as for reactivation [49]. Unfortunately, to the best of our knowledge, specific guidelines on management of psoriatic patients under biological treatment and with concomitant HCV infection are lacking [80]. Therefore, the screening should rely on the rules set for the general population, according to the international guidelines: first, serology (HCV antibody) and then virology (serum HCV-RNA) tests [81]. In case of current HCV infection (HCV antibody reactive and serum HCV-RNA detectable), appropriate counselling and referral to an infectious diseases/hepatology specialist are fundamental steps [82]. If no treatment decisions are made, a prudent and reasonable choice could be a close monitoring of liver function test as well as viremia, namely, each 3–6 months [49]. To our knowledge, there is no study that evaluate the possibility of HCV reactivation during psoriasis systemic treatments. Nowadays, data about Direct-Acting Antiviral Agents (DAAs) treatments in psoriatic patients is still lacking; further studies are needed to investigate this aspect and try to answer to the question [83]. In Table 3, we describe the suspected drug-drug interactions of approved FDA therapies to treat HCV infection and systemic antipsoriatic therapies. This description is based on relevant data, in the public domain, of the University of Liverpool [84]. No data are available of the following antipsoriatic drugs: fumaric acid, certolizumab, brodalumab, guselkumab, ixekizumab, secukinumab, calcipotriene, apremilast, ustekinumab, infliximab, adalimumab, tofacitinib, and golimumab (Table 3).

## 7. Hepatocellular Carcinoma and Psoriasis

Th-17 cells are currently thought to have a bridging role between innate and adaptive immunity and by long a dysfunction in this lineage may support the genesis of autoimmunity and cancer [85, 86]. Despite the universally accepted concept that HCV can provoke HCC [87], the eventual correlation between psoriasis and HCC remains still open. Some cytokines, as IL-6, TNF-*α*, and VEGF, typically overexpressed both in skin and in serum of psoriatic patients have a pivotal role also in HCC [88]. IL-6 is a pleiotropic cytokines involved in chronic inflammation and liver carcinogenesis and found to be related to hepatic function and tumor progression and determine HCC patient survival [89]. TNF-*α* playing an inflammatory role in regulating hepatocyte proliferation and regeneration and its overexpression is related to tumor progression because of released to nonparenchymal cells in HCC [90]. Vascular-endothelial growth factor is highly expressed in HCC as well, a typical hypervascular tumor [91]. The advanced stages of HCC displayed also high levels IL-10 [89], in contrast with psoriasis where IL-10 is usually low [92]. However, Aroucha et al. described that HCC were associated with high TNF-*α*/IL-10 ratio, supposing that the unbalanced production of these cytokines should address to a progression of liver disease in HCV patients [93].

## 8. Nonalcoholic Fatty Liver Disease and Nonalcoholic Steatohepatitis

NAFLD encompasses a continuum spectrum of liver conditions from the simple steatosis to steatohepatitis (NASH), with the risk to evolve to cirrhosis and hepatocellular carcinoma [94]. The prevalence of NAFLD in general population spaces from 10% to 25%, while, among psoriatic patients, the rate is even more, ranging from 17% to 65% depending on the considered studies [95–100]. NASH occurs in 20% of NAFLD patients [96] and globally displays a greater tendency to evolve in psoriatic patients [101]. In fact patients with NAFLD/NASH and psoriasis have higher Psoriasis Area Severity Index (PASI) and C-reactive protein than patients with only psoriasis [98–100]. In add, psoriasis is described to be an important predictor of advanced liver fibrosis [99]. From a pathogenic point of view NAFLD represents the tissue-related manifestation of metabolic syndrome, aspect confirmed by both metabolic profile and epidemiological data of patients with psoriasis and PsA [102]. Recent studies state that metabolome, performed on liver samples, differs, respectively, from healthy controls to NAFLD patients and may be crucial to discriminate NAFLD patients with a tendency to progress to NASH [103]. Assessing the inflammatory background main actors in psoriasis and NAFLD, many proinflammatory cytokines, such as IL-1*β*, TNF-*α*, and IL6, are in common and may create, sustain, and maintain the three stages of NAFLD, namely, inflammation, insulin resistance, and lipid accumulation [104]. TNF-*α* and IL-6 not only drive keratinocyte proliferation and differentiation but also increase insulin resistance and promote proinflammatory cytokines release. Microvascular remodeling in psoriatic skin is conducted mainly by IL-17 and TNF-*α*, that is contemporary due to steatosis and fibrosis of the liver. IL-8, the main neutrophilic chemo attractor, is significantly high and contributes to promote the homing of neutrophils and maintain the proinflammatory microenvironment in both districts. Adiponectins are in the complex altered, to testimony that the lipidic metabolism is deeply perturbed by a chronic systemic status of inflammation. Coherent with the previously discussed data, psoriasis and NAFLD share a common proinflammatory background and may sustain and amplify each other. Empirical evidence arrives from a study that assessed 81 patients with plaque psoriasis, metabolic syndrome, and NAFLD treated for 24 weeks with etanercept, a TNF-blocker, or PUVA therapy. Only the group that undergo etanercept obtained a significant reduction of AST/ALT ratio, C-reactive protein, homeostasis model assessment (HOMA) and an increase to Quantitative Insulin-Sensitivity Check Index (QUICK) [105]. These data seem to highlight the role of inflammation both as a promoter and as a maintainer of NAFLD and psoriasis, leading to the concept that a systemic intervention is needed to contemporary care and limit both conditions.

## 9. Autoimmune Hepatitis

Autoimmune hepatitis (AIH) is a chronic inflammatory liver disease that recognizes the aberrant autoaggressive immunity against self-hepatocyte antigens as first step [95]. Treg ineffective response, selective IgG elevation, and autoreactive T cells are the three mainstays of AIH and together cause the histological evidences of a progressive necroinflammatory interface hepatitis, clinically highlighted by hypertransaminasemia, hypogammaglobulinemia, and circulating autoantibodies [106]. It can coexist with several liver diseases and extrahepatic manifestation, first of all psoriasis among the immune-mediated ones [107]. In fact, as in psoriasis, the involvement of Th17 seems to be crucial in AIH as described by mouse models where the expression of IL-17 was higher than controls in liver specimens and sera; furthermore, the administration of anti-IL-17 neutralizing antibodies markedly improves the hepatic necrosis and decreases the hypertransaminasemia [108].

## 10. Primary Biliary Cirrhosis

Primary Biliary Cirrhosis (PBC) is a chronic inflammatory autoimmune disease primarily involving cholangiocytes of the interlobular bile ducts in the liver with an unexplained geographical variation of prevalence [109]. The diagnosis is made if two of the three criteria are fulfilled: presence of specific-autoantibodies (anti-mitochondrial antibodies, abnormal cholestasis indexes for more than 6 months, chronic nonsuppurative cholangitis followed by progressive bile duct destruction [96, 110]. As summarized in the table, the axis Th1/Th17 is strictly involved in causing and maintaining PBC, as well as psoriasis [111]. Pathogenetic data are confirmed also by epidemiological studies that quantify the prevalence of psoriasis in 13% of PBC patients [109]. Prince et al. described in a case-control study a higher risk of PBC in psoriatic patients than in healthy controls [112]. Weak associations with other autoimmune disease are ascertained [109]. Occasionally PBC may occur together with AIH or PSC leading to the clinical characteristic overlap syndrome strictly coexisting with other autoimmune diseases, especially psoriasis [113, 114]. These data suggest once more the concept of mosaic of autoimmunity, stating that, in genetically predisposed individuals with an abnormal immune response, several autoimmunity disturbances may develop due to the complex interaction between genetic, hormonal, immunological, and environmental factors that are combined in different ways [115].

## 11. Primary Sclerosing Cholangitis

Primary Sclerosing Cholangitis (PSC) is a cholestatic liver and biliary tract disease associated with chronic inflammation of the biliary epithelium, histologically characterized by intra- and extrahepatic biliary structures, and fibrosis [116]. It eventually may evolve in secondary biliary cirrhosis and malignancy [116]. PSC is still a condition characterized by a high rate of misdiagnosis, partially due to the fact that approximately 40–50% of patients are asymptomatic [117]. Due to the fact that the rarity of the condition both prevalence and incidence remain inaccurate, epidemiological data show a strong predilection for male gender [118]. PSC notably is coexistent to autoimmune and autoinflammatory conditions, namely, inflammatory bowel diseases and psoriasis [118] and presents common HLA susceptibility loci [119]. The pathogenesis of PSC remains still unclear; however, the current hypothesis orients to an abnormal response to a gut pathogen in a host with both altered biliary mucosal milieu and a genetic predisposition [118]. The weight of microbiota is still in exam; however, an increased body of evidence may suggest a possible link between skin and gut microbiota and a pivotal role in modulating inflammation [120]. Recently a mice model relates alterations of gut microbiota to imiquimod-induced psoriasis by altering the T cell [121].

## 12. Neutrophilic Cholangitis

Neutrophilic Cholangitis (NC) is an entity recently identified and characterized by a predominantly neutrophilic infiltration of biliary ducts resulting in cholestasis, without sclerotic aspects [122]. No data of incidence and prevalence are present due to the extreme rarity of the disease. The systematic parallel course hypertransaminasemia and psoriasis flares, in a patient negative for viral hepatitis, autoantibodies, and hepatotoxic drug intake, orients to NC. The instrumental confirmation with MRCP that evidences dilatations of intrahepatic bile ducts with or without strictures of the common bile duct; however, histology remains the gold standard for diagnosis of NC [123]. NC appears to be related to neutrophilic-diseases, especially psoriasis [122]. Remarkably, peripheral blood neutrophilia is usually present. As expected, IL-8, the main chemoattractive for neutrophils, has been observed in NC [124] in keratinocytes from skin lesions of psoriasis vulgaris and generalized pustular psoriasis [125] and in synovial lesions of PsA [126], suggesting a key role in the pathogenesis of NC among patients with psoriasis.

## 13. Hepatic Sarcoidosis (HS)

Sarcoidosis is a multisystem disease of unknown aetiology that is seen as a key histological findings noncaseating granulomas. It rarely affects also the liver; hepatic sarcoidosis may present as asymptomatic hepatic granulomas to clinically evident disease with cholestasis or, in advanced cases, cirrhosis and portal hypertension. Occasionally HS may occur together with other inflammatory diseases, namely, psoriasis [127–130].

## 14. Conclusions

The bridge between skin and liver was starting to delineate and psoriasis could be a great pathognomonic example of it. Liver can be affected, directly or indirectly, by psoriasis; consequently, a great attention to the liver profile is mandatory. In accordance with the guidelines, actually almost exclusively psoriatic patients that undergo a systemic therapy are routinely checked for liver affections. Finally, this review aims first to underline the wide spectrum of liver diseases that can co-occur in psoriatic patients and secondary to suggest a routine liver check also in psoriatic patient without a systemic therapy-psoriasis related.

## Figures and Tables

**Table 1:** Comparison of liver toxic substances in psoriatic patients.

Drug	Potential toxicity	Type of Injury	In hepatic insufficiency	Increased risk
Methotrexate	↑	Oxidative stress	NA	NAFLD/Obesity, Leflunomide
Acitretin	↑	Mitochondrial dysfunctions	NA	NA
TNF inhibitors	↑	Autoimmune hepatitis	NA	NA
IL-12/23 blocker	↑	NA	Safe	NA
IL-17 blockers	NA	NA	NA	NA
Cyclosporin	Rare	Oxidative stress	Safe	Obesity
Fructus Psoraleae	↑	Liver lipid metabolism	NA	NA

NA: not applicable.

**Table 2:** Inspired from the American Gastroenterological Association Institute technical review on prevention and treatment of HBVr during immunosuppressive drug therapy.

Drug	Potential disorders for treatment			Risk group	HBVr drug risk estimates (%)
	Group for Research and Assessment of Psoriasis and Psoriatic Arthritis (GRAPPA)	European League Against Rheumatism (EULAR)	European Dermatology Forum (EDF), European Associa- tion for Dermatology and Venereology (EADV), International Psoriasis Council (IPC)		
TNF inhibitors: etanercept, adalimumab, certolizumab, infliximab	Peripheral arthritis, axial disease, enthesitis, dactylitis, psoriasis, nails	Axial, enthesitis, peripheral arthritis, dactylitis	Psoriasis	Moderate	(i) HBsAg positive/anti-HBc positive: 1%–10% (B) (ii) HBsAg negative/anti-HBc positive: 1% (C)

IL-12/23 blocker (Ustekinumab)	Peripheral arthritis, axial disease, enthesitis, dactylitis, psoriasis, nails	Axial, enthesitis, peripheral arthritis, dactylitis	Psoriasis	Moderate	(i) HBsAg positive/anti-HBc positive: 1%–10% (C) (ii) HBsAg negative/anti-HBc positive: 1% (C)

Methotrexate	Peripheral arthritis, dactylitis, psoriasis, nails	Peripheral arthritis, dactylitis	Psoriasis	Low	(i) HBsAg positive/anti-HBc positive: <1% (A) (ii) HBsAg negative/anti-HBc positive: <1% (A)

Corticosteroids	Axial, enthesitis, peripheral arthritis, dactylitis	Axial, enthesitis, peripheral arthritis, dactylitis		High	therapy for ≥4 wk (i) HBsAg positive/anti-HBc positive: >10% (B) (moderate/high dose^*∗*^)* *
				Moderate	therapy for ≥4 wk (i) HBsAg positive/anti-HBc positive: 1–10% (C) (low dose^*∗*^) (ii) HBsAg negative/anti-HBc positive: 1–10% (C) (moderate/high dose^*∗*^)
				Low	therapy for ≥4 wk HBsAg negative/anti-HBc positive: <1% (B) (low dose^*∗*^)
				Low	therapy for ≤1 wk (i) HBsAg positive/anti-HBc positive: <1% (B) (ii) HBsAg negative/anti-HBc positive: <1% (A)

*Note*. Low-risk drug was anticipated to result in HBVr in <1% of cases for all drugs in this category and substantially <1% with most agents; use of a moderate-risk drug was anticipated to result in HBVr in >1% of cases but <10% of cases; and use of a high-risk drug was anticipated to result in HBVr in >10% of cases. Confidence in evidence was graded as follows: (A) high confidence that the estimate lies within group risk boundaries; (B) moderate confidence that the estimate lies within group risk boundaries; (C) little or no confidence that the estimate lies within group risk boundaries. ^*∗*^Glucocorticoids: prednisone (or equivalent): low dose, <10 mg; moderate dose, 10–20 mg; high dose, >20 mg.

**Table 3:** Drug-drug interactions expected of HCV therapies and systemic anti-psoriatic therapies.

	Prednisone	Betamethasone	Methotrexate	Cyclosporine	Acitretin	Etanercept
Daclatasvir	NIE	NIE	PI	NIE	NIE	PWI
Elbasvir/grazoprevir	NIE	NIE	PI	DNC	NIE	PWI
Glecaprevir/pibrentasvir	NIE	NIE	PI	PI	NIE	PWI
Ombitasvir/paritaprevir/ritonavir	PI	PI	NIE	PI	NIE	PWI
Ombitasvir/paritaprevir/ritonavir/dasabuvir	PI	PI	NIE	PI	NIE	PWI
Simeprevir	PI	PI	NIE	DNC	NIE	PWI
Sofosbuvir	NIE	NIE	NIE	NIE	NIE	PWI
Sofosbuvir/ledipasvir	NIE	NIE	NIE	NIE	NIE	PWI
Sofosbuvir/velpatasvir	NIE	NIE	PI	NIE	NIE	PWI
Sofosbuvir/velpatasvir/voxilaprevir	NIE	NIE	DNC	DNC	NIE	PWI

DNC: do not coadminister; NIE: no interaction expected; PI: potential interaction; PWI: potential weak interaction.
